# Risk Stratification and Management of Intermediate- and High-Risk Pulmonary Embolism

**DOI:** 10.3390/jcm13185583

**Published:** 2024-09-20

**Authors:** Sanaullah Mojaddedi, Javairia Jamil, Daniel Bishev, Kobina Essilfie-Quaye, Islam Y. Elgendy

**Affiliations:** 1 College of Medicine, University of Central Florida, Graduate Medical Education, Orlando, FL 32827, USA; sanaullah.mojaddedi@hcahealthcare.com (S.M.); kobina.essilfiequaye@hcahealthcare.com (K.E.-Q.); 2 Internal Medicine Residency Program, HCA Florida North Florida Hospital, Gainesville, FL 32605, USA; 3 College of Medicine, Gulf Medical University, Ajman P.O. Box 4184, United Arab Emirates; 4 Division of Cardiovascular Medicine, Gill Heart Institute, University of Kentucky, Lexington, KY 40536, USA

**Keywords:** pulmonary embolism, venous thromboembolism, systemic thrombolysis, catheter-directed thrombolysis, catheter-directed embolectomy

## Abstract

Acute pulmonary embolism (PE) is a leading cause of mortality. Not only is PE associated with short-term mortality, but up to ~20% of patients might suffer from long-term consequences such as post-PE syndrome and chronic thromboembolic pulmonary hypertension. Current risk stratification tools poorly predict those who are at risk for short-term deterioration and those who develop long-term consequences. Traditionally, systemic thrombolysis has been considered the first-line therapy for patients with high-risk PE without contraindications; however, it comes with the risk of major bleeding (notably intracranial hemorrhage). The use of catheter-directed interventions (embolectomy or thrombolysis) has been increasing owing to their low bleeding risk; however, randomized trial data supporting their efficacy in improving clinical outcomes are limited. In this review, we highlight the evidence supporting the available advanced therapies for high- and intermediate-risk PE and summarize the ongoing trials which are evaluating these therapies.

## 1. Introduction

Acute pulmonary embolism (PE) is the third most common acute cardiovascular syndrome and a leading cause of death [1]. The annual PE incidence in the United States is 39–115 per 100,000 people [1], with an estimated 300,000 deaths annually [2]. The incidence and mortality rates related to PE have remained relatively stable between 1999 and 2018 [3]. Despite the widespread use of imaging modalities to diagnose PE such as CTPA, the current risk stratification tools are not accurate in predicting short-term mortality and long-term consequences. PE can be classified based on the presentations to: (i) low risk (hemodynamically stable patients without evidence of right ventricular dysfunction); (ii) intermediate-low risk (hemodynamically stable patients with either right ventricular dysfunction or elevated cardiac biomarkers), (iii) intermediate-high risk (hemodynamically stable patients with both right ventricular dysfunction and elevated biomarkers), and (iv) high-risk patients (hemodynamic instability) [4]. Traditionally, systemic thrombolysis has been considered first-line therapy for high-risk PE, but due to high rates of major bleeding, specifically intracranial hemorrhage, alternative therapies such as catheter-directed interventions are becoming more widely used. In this review, we discuss the risk stratification tools and emerging management options for intermediate- and high-risk PE.

## 2. Risk Factors for Pulmonary Embolism

Risk factors for PE are categorized as major transient, minor transient, non-malignant persistent, malignant, and underlying hypercoagulable states. Major transient risk factors include bed rest >3 days, and fractures; minor transient risk factors include central venous lines, long-haul travel, and oral contraceptive therapy; non-malignant persistent risk factors include inflammatory bowel disease and active autoimmune disease, whereas malignancy and hypercoagulable states are separate risk factors [4,5,6].

Venous thromboembolism (VTE) triggered by a major transient/reversible risk factor has an annual event rate of 3.3% (95% CI, 2.8%–3.9%) recurrence after therapy discontinuation [7]. Conversely, for VTEs caused by persistent and progressive risk factors (e.g., metastatic cancer), the risk of recurrence is 20.7% over 12 months (95% CI, 15.6%–25.8%) after discontinuing therapy [8]. Patients without significant transient/reversible risk factors for VTE (previously classified as unprovoked) face an intermediate risk of recurrence after completing anticoagulation therapy [9].

It is important to categorize patients who developed VTE according to their risk factors to determine the length of therapy and risk of recurrence (Table 1). Patients with hereditary thrombophilia, such as confirmed deficiencies of antithrombin, protein C, or protein S, are often candidates for indefinite anticoagulant treatment following an initial episode of PE without a major reversible risk factor [4].

## 3. Pathophysiology

PE, particularly saddle PE, leads to the sudden obstruction of the pulmonary arteries, primarily leading to right ventricular (RV) failure secondary to acute pressure overload [4]. When significant portions (30–50%) of the pulmonary bed are obstructed, there is a significant increase in pulmonary arterial pressure and elevated pulmonary vascular resistance [11]. The obstruction and resultant hypoxic vasoconstriction, combined with the release of neurohumoral factors such as serotonin, thromboxane A2, and histamine, escalate pulmonary vasculature resistance, imposing a substantial afterload on the RV [9].

**Figure 1:**
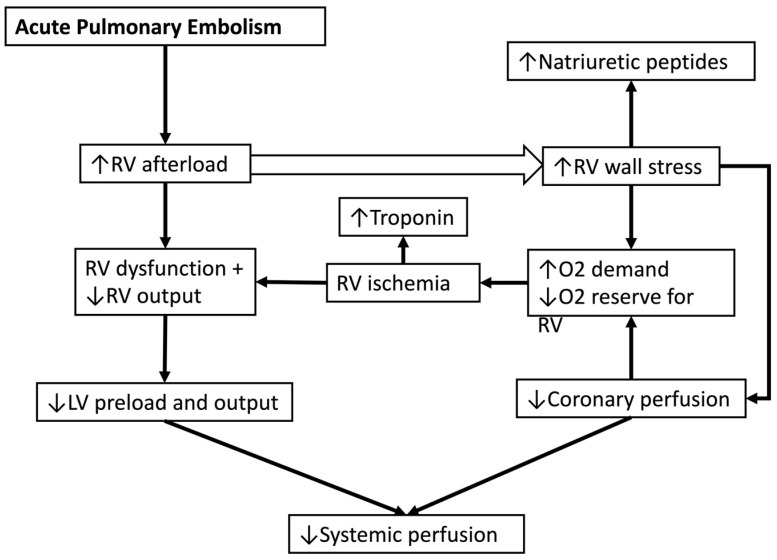
Pathophysiology of shock in acute pulmonary embolism. RV: right ventricle, LV: left ventricle, O2: oxygen.

The acute RV pressure overload induces RV dilation and hypokinesis, contributing to tricuspid regurgitation and reduced cardiac output. As the RV dilates, the interventricular septum shifts towards the left ventricle (LV), impairing LV diastolic filling and reducing systemic blood pressure [4]. The strain on the RV myocardium increases myocardial oxygen demand, with subsequent decreased coronary perfusion due to obstructive shock, leading to ischemia, decreased systemic perfusion, and potential RV infarction (Figure 1) [12].

Additionally, the mismatch between ventilation and perfusion causes hypoxemia. In severe cases, the pressure gradient may reverse through a patent foramen ovale, resulting in right-to-left shunting and worsening hypoxemia. These pathophysiological changes rapidly progress towards obstructive shock if left untreated, necessitating prompt medical intervention to restore hemodynamic stability and prevent fatal outcomes [13].

## 4. Risk Stratification

The cornerstone of PE diagnosis remains imaging, namely with computed tomography pulmonary angiography (CTPA) [14]. Bedside transthoracic echocardiography (TTE) can visualize right ventricular dysfunction and suffice for the initiation of pulmonary reperfusion therapy in a hemodynamically unstable patient [14]. One meta-analysis including 1249 patients found that echocardiographic evidence of RV dysfunction was associated with 2.4-fold increased odds of all-cause mortality (OR 2.4, 95% CI, 1.3–4.3) [15]. A recent study found that patients with intermediate-risk PE presenting with McConnell’s sign had increased odds of normotensive shock (OR 8.4, 95% CI, 1.7–40.5) [16].

Earlier scoring systems such as the Pulmonary Embolism Severity Index (PESI) and simplified PESI (sPESI) have been used to classify patients as low risk or high risk. PESI utilizes 10 clinical markers, each with their own point value, to divide patients into classes I-V. These markers include the male sex (+10 points), chronic heart failure (+10 points), chronic pulmonary disease (+10 points), tachycardia >110 bpm (+20 points), tachypnea >30 breaths per min (+20 points), temperature >36 °C (+20 points), arterial oxyhemoglobin saturation <90% (+20 points), cancer (+30 points), systolic blood pressure <100 mmHg (+30 points), and acute encephalopathy (+60 points) [17]. The different classes are classified according to 30-day mortality risk: classes I–II (0–85 points) are considered low-risk, with a 30-day mortality up to 3.5%; class III (86–105 points) is moderate risk with a 3.2–7.1% 30-day mortality; class IV (106–125 points) carries a high 30-day mortality risk with a value up to 11.4%; and lastly class V (>125 points) is considered a very high mortality risk, with up to 24.5% 30-day mortality [4]. sPESI uses age >80, a history of chronic lung disease or heart failure, history of cancer, systolic blood pressure <100 mmHg, pulse rate >110 beats/min, and SaO_2_ < 90% for risk stratification, with each criteria giving 1 point to the patient [18]. Patients with a score of zero are classified as low risk while scores of ≥1 are considered high-risk PE [18]. The sPESI score performs well in identifying low-risk PE patients. One meta-analysis with over 50,000 patients showed that a low sPESI was associated with a 2.1% incidence of all-cause mortality [19]. Notably, both the PESI scores do not take into consideration the RV function, which is a strong predictor of potential hemodynamic compromise with worse outcomes [4]. Another limitation is the inclusion of several vitals and comorbidities which can be attributed to other clinical conditions like sepsis, heart failure, and chronic obstructive pulmonary disorder exacerbation.

The 2019 European Society of Cardiology (ESC) guidelines for the diagnosis and management of acute PE use markers of RV function (cardiac troponin, BNP, NT-pro-BNP) to risk stratify acute PE into low, intermediate-low, intermediate-high, and high-risk patients. The intermediate-low group is defined as sPESI ≥1 with or without RV function markers, the intermediate-high group as sPESI ≥1 with elevated troponins and positive RV dysfunction imaging findings, and the high-risk as sPESI ≥1 with elevated troponins, positive RV dysfunction imaging findings, and hemodynamic instability (Table 2) [4].

However, this classification does not predict clinical deterioration in the intermediate-high-risk group who might further deteriorate and develop hemodynamic compromise. Some authors have classified this group of patients as “normotensive shock” (i.e., systolic blood pressure ≥ 90 mmHg with a cardiac index ≤2.2 L/min/m^2^). In one study of 384 patients with intermediate risk PE who underwent mechanical thrombectomy, almost a third had normotensive shock. A composite shock score including elevated cardiac troponins, elevated natriuretic peptides, RV dysfunction, saddle PE, concomitant DVT, and tachycardia was developed. The prevalence of normotensive shock increased with a larger number of components of the composite shock score: a score of 0 had a 0% prevalence of normotensive shock, whereas a score of 6 had a normotensive shock prevalence of 58.3% (OR 5.8, 95% CI, 2.0–17.0) [20]. This composite shock score was also found to predict worse in-hospital outcomes among patients with intermediate-risk PE in one retrospective single-center study [21]. Future prospective multicenter studies are needed to validate this composite shock score in a larger population of intermediate-risk PE patients.

## 5. Long-Term Complications

Long PE syndrome is a common entity comprising persistent dyspnea or poor physical performance over several months to years following acute PE. These patients may develop chronic thromboembolic disease or chronic thromboembolic pulmonary hypertension. The majority of PE survivors restore their pulmonary artery bed patency within the first few months following the acute insult [22]. However, cohort studies have revealed that these symptoms frequently persist for 6 months to 3 years [4]. One meta-analysis of 26 studies with 3671 patients showed that the prevalence of persistent RV dysfunction after 3 months was 18.1% and the prevalence of at least mild functional impairment (NYHA II-IV) was 33.2%. There was no difference in the incidence of these functional impairments based on the initial modality of therapy (thrombolysis versus anticoagulation alone) [23]. A recent cohort study in Canada followed 100 PE survivors over one year; 46.5% of patients had reduced maximal aerobic capacity, defined as a peak oxygen consumption <80% on cardiopulmonary exercise testing [24]. Of note, these patients had pulmonary function tests and echocardiographic findings largely within normal limits at follow-up. Some factors have been demonstrated to be associated with reduced functional exercise capacity and quality of life such as obesity, prior lung disease, the female sex, higher pulmonary artery systolic pressures on a day-10 echocardiogram, and higher main pulmonary artery diameter on the baseline CTPA [25]. The development of long PE syndrome appears unrelated to the degree of hemodynamic compromise at the time of the initial event, and can include chronic thromboembolic pulmonary hypertension and chronic thromboembolic disease without pulmonary hypertension. In one study of 20 survivors with intermediate-high and high-risk PE, there was no association between exercise impairment and persistent RV dysfunction [26]. It remains unclear whether advanced therapies besides systemic anticoagulation would reduce the risk of developing long PE syndrome.

## 6. Management Strategies

Systemic anticoagulation is the cornerstone of PE therapy, with advanced therapies including thrombolytics and embolectomy reserved for the high-risk and intermediate-risk PE. Certain cases may require adjunctive therapy with extracorporeal mechanical circulatory support (ECMO), which provides immediate support for acute circulatory collapse but does not treat the PE.

### 6.1. Systemic Thrombolysis

The benefits of this treatment modality include availability and applicability without additional training. Unfortunately, PE patients often have absolute or relative contraindications to systemic thrombolysis. A meta-analysis of 16 trials of 2115 patients (of which 8 trials enrolled intermediate-risk PE patients) showed that systemic thrombolysis reduced all-cause mortality (OR 0.5, 95% CI, 0.3–0.9, number needed to treat = 59), but with the expense of excess intracranial hemorrhage (OR 4.6, 95% CI, 1.8–12.0, number needed to harm = 78), especially in older patients (i.e., >65 years) (OR 3.1, 95% CI, 2.1–5.6, number needed to harm = 11) [27]. The PEITHO trial compared tenecteplase versus a placebo in 1005 intermediate-high-risk PE patients, which showed a decreased incidence of death or hemodynamic decompensation in the tenecteplase group (OR 0.4, 95% CI, 0.2–0.9, *p* = 0.02) but this was associated with elevated risk of extracranial bleeding (6.3% vs. 1.2%, *p* = 0.001) and stroke (2.4% vs. 0.1%, *p* = 0.003) [28]. Based on the findings of this trial, systemic thrombolysis is not recommended among patients with intermediate PE (i.e., without hemodynamic instability).

### 6.2. Catheter-Directed Thrombolysis

This is a minimally invasive procedure that involves delivering lower doses of thrombolytic therapy locally into the pulmonary vasculature [29]. This can be carried out using ultrasound-assisted catheter-directed thrombolysis (UA-CDT), where ultrasonic pressure waves are emitted along the catheter which improve the delivery of the thrombolytic agent, in vitro or using multi-hole catheters such as Unifuse and Cragg-McNamara (Table 3). This allows for the delivery of a smaller dose over a shorter period. The ULTIMA trial, which enrolled 59 patients with intermediate-risk PE, showed that UA-CDT was superior in reversing the RV/LV ratio at 24 h (mean decrease of 0.3 ± 0.0 vs. 0.0 ± 0.2, *p* < 0.001) without increasing bleeding complications, compared with systemic anticoagulation alone [30]. The SEATTLE 2 trial was a prospective single-arm multicenter trial of 150 patients that also showed US-CDT decreasing RV dilatation (mean difference, −0.4; *p* < 0.0001) and reduced pulmonary artery systolic pressure (51.4 mmHg vs. 36.9 mmHg, *p* < 0.0001) at 48 h post-procedure [31]. No patients developed ICH in the UA-CDT arm in the ULTIMA or SEATTLE 2 trials.

In the OPTALYSE trial, the investigators attempted to find the optimal dosing of tissue plasminogen activator (tPA) and the delivery duration during UA-CDT. They enrolled 101 patients with intermediate-risk PE and administered four different dose duration variations ranging from 4 mg per lung over two hours to 12 mg per lung over six hours. One patient developed ICH, and thus the trial concluded that shorter durations with smaller tPA doses were associated with improved RV function and a reduced clot burden [33].

The SUNSET sPE trial examined 81 patients with intermediate-risk PE and randomized them to US CDT or standard CDT. This trial found that patients in both treatment arms had similar reductions in thrombus reduction (*p* = 0.76) [34]. In one large retrospective study of 39,430 patients from the National Inpatient Sample between 2016 and 2020, there was no difference in the incidence of in-hospital mortality (OR 0.75, 95% CI, 0.5–1.1, *p* = 0.1) and major bleeding between US-CDT and standard CDT [35].

A large meta-analysis of 45 studies, including 18 randomized control trials (RCT) and 28 observational studies including 81,705 patients with intermediate- and high-risk PE (of which 20 studies exclusively included intermediate-risk PE patients), found that compared with anticoagulation, CDT was associated with lower mortality but higher bleeding risk (OR 0.6, 95% CI, 0.4–0.8 and OR 1.8, 95% CI, 1.1–3.1, respectively). CDT was found to have a better safety profile when compared with systemic therapy [36]. The findings were mostly consistent in the subgroup analysis of the intermediate-risk PE patients.

### 6.3. Catheter-Directed Embolectomy

This is another minimally invasive procedure using larger-bore catheters to extract the thrombus instead of delivering thrombolytic therapy. Catheter-directed embolectomy (CDE) is performed either through suction/aspiration or extraction/disruption. Currently, there are no RCTs comparing CDE to anticoagulation or CDT.

The FLARE study was a single-arm, multicenter, prospective trial analyzing the effectiveness of percutaneous mechanical thrombectomy in treating patients with intermediate-risk PE. The study enrolled 106 patients across 18 U.S. sites and treated patients with the FlowTriever system. The results of the trial revealed that mechanical thrombectomy improved the RV/LV ratio, with minimal major bleeding (mean RV/LV ratio reduction of 0.4, *p* < 0.0001) at 48 h. Furthermore, the advantages of CDE include immediate thrombus removal, the absence of fibrinolytic complications, and a reduced need for post-procedural critical care [37].

The FLASH registry analyzed 800 PE patients (77% with intermediate-risk PE) from a U.S. national registry to evaluate the safety and effectiveness of mechanical thrombectomy for intermediate- and high-risk PE in a real-world population. A total of 14 major adverse events (1.8%) were observed at 48 h with a 0.8% 30-day all-cause mortality and only three (0.4%) intraprocedural major adverse events. Immediately following thrombectomy, the cardiac index improved by a mean of 0.3 L/min/m^2^ (mean change of 19.0%, *p* < 0.0001) and the RV/LV ratio decreased (1.2 ± 0.4–1.0 ± 0.3, *p* < 0.0001) at 48 h [38]. 

Indigo is a smaller caliber CDE catheter using high-velocity suction to perform thrombectomy. This device was studied in a single-arm prospective, multicenter study named EXTRACT-PE, which included 119 patients. This study showed a mean RV/LV ratio reduction at 48 h from a baseline of 0.4 (95% 0.4–0.5, *p* < 0.0001), with a low adverse event rate. The trial did not include patients in shock and thrombolytics were avoided in 98.3% of patients [23].

In an analysis of the Nationwide Readmission Database between the years 2016 and 2019 and including 3216 patients, there was no difference in the incidence of all-cause mortality (OR 1.3, 95% CI, 1.0–1.7), ICH (OR 1.6, 95% CI, 0.8–3.3), and non-ICH major bleeding (OR 1.2, 95% CI, 0.9–1.6) between CDT and CDE [39]. In another analysis of the same database, there was an inverse relationship between a larger procedure volume with CDT or CDE (>12 procedures annually) and in-hospital mortality, length of stay, and cost [40]. The REAL-PE study included >4000 patients treated with US-CDT or mechanical thrombectomy, comparing the risk of bleeding through direct laboratory analysis and transfusion administration documentation which showed an increased risk of bleeding with mechanical thrombectomy (17.3% vs. 12.4%, *p* = 0.002) [41]. However, the findings should be interpreted in the context of a retrospective observational study, and the reason for the allocation of one therapy versus the other could not be ascertained.

### 6.4. Surgical Thrombectomy

Surgical thrombectomy is not commonly performed, and mainly reserved for high-risk PE patients who have contraindications to thrombolysis or for unstable patients with systemic thrombolysis despite being in highly experienced centers. Surgical thrombectomy has been linked with high in-hospital mortality in several single-center series [42,43,44]. One meta-analysis consisting of eight observational studies with 1403 patients compared CDT to surgical- and catheter-directed thrombectomy and showed that CDT had lower-in hospital mortality (RR 0.6, 95% CI, 0.4–0.9, *p* = 0.01), with similar rates of major bleeding (*p* = 0.6), stroke (*p* = 0.4), and atrial fibrillation (*p* = 0.7) [45]. These findings should be considered in light of retrospective observational studies, which are limited by selection and ascertainment biases.

### 6.5. Mechanical Circulatory Support

Critically ill patients with PE occasionally experience circulatory collapse requiring mechanical circulatory support (MCS). This can be achieved with veno-arterial extracorporeal membrane oxygenation (VA-ECMO). The VA-ECMO oxygenates blood drawn from the right atrium through an external oxygenation membrane and returns it into an artery. This allows complete the bypass of the cardiopulmonary system as a bridge to more permanent therapy. The use of VA-ECMO as a “bridge to recovery” by only treating the patient with anticoagulation is not supported by randomized clinical trial data [4], and is based on single-center experiences. Because patients who received VA-ECMO in these studies were high-risk patients, the mortality was considerably high [46,47]. MCS can be utilized as a bridge to another advanced therapy modality such as percutaneous embolectomy, or surgical embolectomy [48].

## 7. Selection of Therapy

Given the paucity of high-quality RCT data supporting the use of most of these advanced therapies, there are controversies in the level of recommendation for these therapies between the guidelines (Table 4). The choice of therapy often is related to the institutional expertise and availability of advanced therapies. Pulmonary Embolism Response Teams (PERTs) are multidisciplinary teams of healthcare professionals with expertise related to the management of PE. There have been a number of single-center experiences which have showed that the implementation of PERT is linked with the increased use of advanced therapies (particularly catheter-based interventions), and some of these studies showed improved outcomes after the implementation of PERT [49,50,51,52].

## 8. Future Directions

Future research on the management of PE (particularly intermediate-risk PE) should focus on identifying better risk stratification models, integrating biomarkers, and improved imaging techniques. Articifical intellignece could potentially be trained to improve imaging findings. Since prior trials testing catheter-based interventions have only evaluated surrogate outcomes; there are a number of ongoing trials evaluating the clinical outcomes with these catheter-directed therapies for patients with intermediate- and high-risk PE (Table 5). One trial (Hi-PEITHO) is specifically investigating whether CDT will improve long-term functional outcomes among patients with intermediate-risk PE. Additionally, exploring the implementation of comprehensive care pathways that involve multidisciplinary teams can optimize patient management. Conducting long-term studies on the chronic effects of PE and the development of rehabilitation programs is essential to improve patient quality of life and recovery. Emphasizing patient education and adherence to treatment plans will also play a vital role in reducing recurrence rates and enhancing overall outcomes. Investing in healthcare infrastructure to support these innovations is necessary to ensure broad accessibility and effective implementation.

## 9. Conclusions

PE remains a leading cause of death. Our current risk stratification tools do not reliably predict clinical deterioration especially among intermediate risk patients. The currently available risk stratification tools also poorly predict who may benefit from advanced therapies. Traditionally, systemic thrombolysis has been considered the first-line therapy for high-risk PE based on older RCTs but with the expense of an elevated risk of ICH. Catheter-based interventions have shown promising results in improving surrogate outcomes, and there are several ongoing trials to test their efficacy in improving clinical outcomes.

## Figures and Tables

**Table 1:** Categorization of risk factors for VTE based on risk of recurrence [4].

Estimated Risk for Long-Term Recurrence	Risk Factors	Examples
Low (<3% per year)	Major transient/reversible factors associated with >10-fold increase for initial VTE event	Surgery with general anesthesia >30 min Confined to bed in hospital with only bathroom privileges ≥3 days Trauma with fractures
Intermediate (3–8% per year)	Transient/reversible factors associated with ≤10-fold increase for initial VTE event	Minor surgery with general anesthesia <30 min Hospital admission with an acute illness <3 days Leg injury (without fracture) associated with reduced mobility Estrogen therapy, pregnancy, or puerperium Long-haul flight ≥6 h [10]
	Non-malignant persistent risk factors	Inflammatory bowel disease Active autoimmune disease
	No identifiable risk factor	
High (>8% per year)		Active cancer with ≥1 previous episodes of VTE in the absence of a major transient/reversible risk factor Antiphospholipid antibody syndrome

**Table 2:** The classification of pulmonary embolism severity and the risk of early (in-hospital or 30-day) mortality [4].

	Indicators of Risk			
Early Mortality Risk	Hemodynamic Instability	Clinical Parameters of PE Severity and/or Cormorbidity; PESI Class III-V or sPESI ≥1	RV Dysfunction on TTE or CTPA	Elevated Cardiac Troponin Levels
High	+	+	+	+
Intermediate-high	-	+	+	+
Intermediate-low	-	+	One or more positive	
Low	-	-	-	Assessment optional; negative

CTPA: computed tomography pulmonary angiography, TTE: transthoracic echocardiography, PESI: pulmonary embolism severity index, sPESI: simplified pulmonary embolism severity index.

**Table 3:** Treatment options for intermediate- and high-risk pulmonary embolus [32].

Device	Sheath	Technique	Trial and Indications	Advantages	Disadvantages
Catheter-Directed Thrombolysis					
EKOS Endovascular System	5.2 Fr	High-frequency, low-power ultrasound waves aim to disrupt the clot, allowing the lytic agent to penetrate clot with a lower dose requirement	ULTIMA	Ultrasound may break fibrin strands, allowing the improved penetration of the thrombolytic agent Lower volumes of the lytic agent	Benefit from ultrasound unclear Costlier compared to CDT
			- 20 mg tPA - 0.3 difference in RV/LV ratio - 0% major bleeding		
			SEATTLE II		
			- 24 mg of tPA - 0.42 difference in RV/LV ratio - 10% major bleeding, no ICH		
			OPTALYSE-PE		
			- 4–12 mg of tPA for 2–6 h - 0.3–0.4 difference in RV/LV ratio - 1 episode of ICH in the arm of 12 mg for over 6 h - Overall risk of bleeding of 4%		
Non-Ultrasound devices (Unifuse, Cragg-McNamara)	4, 5 Fr	Multi-hole infusion catheters are used for the controlled, selective infusion of thrombolytic medication into the vasculature	SUNSET sPE	Pressure-response technology provides an even distribution of the lytic agent No guidewire (Cragg-McNamara), allowing for larger volume of the lytic agent	Typically requires 12–24 h for thrombolytic infusion
			- 81 patients with intermediate-risk PE - Ultasound-assisted thrombolysis vs. non-US assisted or SDCT - Both groups had similar reduction in thrombus reduction (*p* = 0.76)		
Suction/Aspiration Thrombectomy					
Indigo Aspiration System	4, 6, 8 Fr	High-velocity vacuum suction catheter	EXTRACT PE	Flexibility for placement in segmental branches	No mechanism for recirculation Luminal diameter limits the volume of thrombus aspiration
			- 0.43 reduction in the RV/LV ratio after 48 h - Small caliber - Only studied patients with submassive PE		
FlowTriever System	20 Fr	A large-aspiration guide catheter; the device has three self-expanding nitinol disks unsheathed to disrupt and aspirate the clot	FLARE	Ability to remove large volumes of thrombus quickly Aspirated blood can be filtered and reinfused	Size/rigidity limits access to pulmonary artery branches Large-bore access required
			- No tPA required - Major bleeding rate of 0.9%		
Surgical Therapies					
Embolectomy	-	Thoracostomy or sternotomy		Contraindications to thrombolysis or hemodynamic instability despite systemic anticoagulation	High risk of complications
Mechanical circulatory support	VA-ECMO or Impella RP		- Bridge to more permanent therapy - Mortality benefit for high-risk PE patients when used in conjunction with surgical embolectomy	Offloads RV, bypasses the pulmonary circulation Recovery may occur with anticoagulation	High bleeding risk with thrombolysis Large-bore arterial cannula required

**Table 4:** Major recommendations for high-risk PE from 2019 European Society of Cardiology (ESC) [4], 2021 American Heart Association (AHA) [53], and 2018 Pulmonary Embolism Response Team (PERT) [54] guidelines.

Guidelines	Recommendation	Class of Recommendation	Level of Evidence
2019 ESC	Systemic thrombolysis	I	B
	Surgical embolectomy (failed systemic thrombolysis/contraindication)	I	C
	Cather-based therapy (failed systemic thrombolysis/contraindication)	IIa	C
	ECMO	IIb	C
2021 AHA	Systemic thrombolysis	IIa	B
	Surgical embolectomy (failed systemic thrombolysis/contraindication)	IIb	C
	Cather-based therapy (failed systemic thrombolysis/contraindication)	IIb	C
2018 PERT	Systemic thrombolysis	Expert opinion	Expert opinion
	Surgical embolectomy (failed systemic thrombolysis/contraindication)		
	Cather-based therapy (failed systemic thrombolysis/contraindication)		
	MSC (refractory shock or cardiac arrest)		

**Table 5:** Ongoing trials studying advanced therapies for PE.

Trial	Clinical Trial Registration	Planned Sample Size	Intervention Arm	Control Arm	Primary Outcome
HI-PEITHO	NCT04790370	406	Ekos Catheter plus anticoagulation	Anticoagulation alone	Within 7 days of randomization: PE-related mortality, PE recurrence, cardiorespiratory decompensation, or collapse
PE-TRACT	NCT05591118	500	Catheter-directed therapy plus anticoagulation	Anticoagulation alone	Day 7: incidence of major bleeding Month 3: PVO2 measured during CPET Month 12: NYHA class
STORM-PE	NCT05684796	100	Indigo Aspiration System, computer-assisted vacuum thrombectomy plus anticoagulation	Anticoagulation alone	Change in RV/LV ratio at 48 h after original therapy as assessed by CTPA 48 h post-randomization
PEERLESS	NCT05111613	550	FlowTriever System	Catheter-directed thrombolysis	Assessed at hospital discharge or 7 days post-procedure Constructed as a hierarchal win ratio: (1) all-cause mortality, (2) ICH, (3) major bleeding, (4) clinical deterioration and/or escalation to bailout, and (5) ICU admission and LOS
PEERLESS 2	NCT06055920	1200	FlowTriever System plus anticoagulation	Anticoagulation alone	Hierarchical composite win ratio of (1) all-cause mortality, (2) clinical deterioration, (3) all-cause hospital readmission, (4) bailout therapy, and (5) mMRC dyspnea score of ≥1 at 48 h visit

PVO2: peak oxygen consumption, CPET: cardiopulmonary exercise testing, NYHA: New York Heart Association, CTPA: computed tomography pulmonary angiography, ICH: intracranial hemorrhage, ICU: Intensive Care Unit, LOS: Length of Stay, mMRC: Modified Medical Research Council.
